# How genetic risk contributes to autoimmune liver disease

**DOI:** 10.1007/s00281-022-00950-8

**Published:** 2022-06-01

**Authors:** David Ellinghaus

**Affiliations:** Institute of Clinical Molecular Biology (IKMB), Kiel University and University Medical Center Schleswig-Holstein, Rosalind-Franklin-Str. 12, 24105 Kiel, Germany

**Keywords:** Autoimmune hepatitis, Primary biliary cholangitis, Primary sclerosing cholangitis, Genome-wide association studies (GWAS), Genome-wide meta-analyses (GWMA), HLA, Non-HLA, Polygenic risk score, Mediated expression score regression

## Abstract

Genome-wide association studies (GWAS) for autoimmune hepatitis (AIH) and GWAS/genome-wide meta-analyses (GWMA) for primary biliary cholangitis (PBC) and primary sclerosing cholangitis (PSC) have been successful over the past decade, identifying about 100 susceptibility loci in the human genome, with strong associations with the HLA locus and many susceptibility variants outside the HLA locus with relatively low risk. However, identifying causative variants and genes and determining their effects on liver cells and their immunological microenvironment is far from trivial. Polygenic risk scores (PRSs) based on current genome-wide data have limited potential to predict individual disease risk. Interestingly, results of mediated expression score regression analysis provide evidence that a substantial portion of gene expression at susceptibility loci is mediated by genetic risk variants, in contrast to many other complex diseases. Genome- and transcriptome-wide comparisons between AIH, PBC, and PSC could help to better delineate the shared inherited component of autoimmune liver diseases (AILDs), and statistical fine-mapping, chromosome X-wide association testing, and genome-wide in silico drug screening approaches recently applied to GWMA data from PBC could potentially be successfully applied to AIH and PSC. Initial successes through single-cell RNA sequencing (scRNA-seq) experiments in PBC and PSC now raise high hopes for understanding the impact of genetic risk variants in the context of liver-resident immune cells and liver cell subpopulations, and for bridging the gap between genetics and disease.

## Introduction

The liver can be affected by three leading forms of complex autoimmune liver diseases (AILDs), in which the immune system attacks different sites in the liver: autoimmune hepatitis (AIH), primary biliary cholangitis (PBC), and primary sclerosing cholangitis (PSC). The exact pathogenesis of these diseases is poorly understood, and available therapeutic approaches are only partially effective. AIH is a rare, chronic progressive disease with a prevalence of approximately 4–42 per 100,000 depending on geographical location [1]. It is characterized by elevated levels of serum transaminases and immunoglobulin G (IgG), inflammatory liver histology, the presence of autoantibodies, and the absence of markers for viral hepatitis [2]. AIH occurs predominantly in middle-aged women but can affect all age groups of both sexes [3]. The exact cause of AIH is unknown, although loss of tolerance against liver antigens is thought to be the major pathophysiologic mechanism caused by an unknown trigger in individuals with a genetic susceptibility [4]. Concordance of AIH and familial clustering of AIH were found in twins, with an estimated pair-wise concordance rate in monozygotic twins of 8.7%, suggesting genetic risk factors for AIH [5]. PBC is a rare disease in which a cycle of immune-mediated damage to biliary epithelial cell, cholestasis, and progressive fibrosis over time can lead to end-stage biliary cirrhosis. PBC more commonly affects women, with a prevalence of about 4–58 per 100,000 people [6], and is characterized by autoimmune destruction of small to medium size intrahepatic bile ducts and the presence of anti-mitochondrial antibodies (AMAs), which are present in > 90% of cases [7, 8]**.** The onset of the disease is thought to be due to the interaction of environmental triggers and a genetic predisposition. Genetic risk is consistent with other complex autoimmune diseases, with an estimated sibling relative risk of 10.5 [9] and a pairwise concordance rate in identical twins of 63%, which is among the highest reported in autoimmune diseases [10]. PSC is a rare disease characterized by multifocal biliary strictures and progressive liver disease. In PSC, there is autoimmune damage to the medium to large bile ducts leading to concentric and obliterative fibrosis and structuring [11]**.** The prevalence of PSC is approximately 10 per 100,000 [12]. The most common positive autoantibodies are perinuclear antineutrophil autoantibodies (pANCA), which are detected in about 80% of patients but are not specific enough for diagnosis [13, 14]. Unlike AIH and PBC, men are more commonly affected by PSC than women [15]. First-degree relatives of patients with PSC have an 11.5-fold increased risk of PSC [16].

AIH, PBC, and PSC are not yet curable. Progression of the disease, especially in PSC, often leads to liver transplantation or death. AIH is usually treated by regulating the immune system with steroid and thiopurine-based treatments [17], which is why therapy in AIH is often associated with significant side effects [4]. In many cases, the drugs can permanently suppress AIH and allow patients to live a normal life. If the disease progresses to cirrhosis despite consistent therapy, liver transplantation is the only option. Two drugs, the natural bile acid ursodeoxycholic acid (UDCA) and the semi-synthetic bile acid obeticholic acid (OCA), are approved and mainly used for the treatment of PBC. Long-term therapy with UDCA is often successful if started early [18], but many patients respond poorly to both agents, putting them at risk of progressive liver disease [19]. Therapy of PSC is also carried out with UDCA, but in contrast to PBC, the success of therapy in PSC is rather limited. In certain circumstances, liver transplantation is the only therapeutic option. Biologics, such as anti-tumor necrosis factor alpha (TNF-α) or B cell-depleting antibodies [20], and many new drugs are currently being investigated for the treatment of AILDs [21]. Unfortunately, there are still no promising drugs that target the (unknown) key pathogenic processes in the early phase of disease progression [22]. Of central importance is the improvement of risk stratification strategies, which requires in-depth, longitudinal phenotyping of patients using multi-omics data analysis. The different course of the disease and the different response of patients to treatment could also be related to the heterogeneous genetic background of individual patients, which translates into a heterogeneous clinical phenotype [23]**.** Elucidating the genetic architecture of AIH, PBC, and PSC is likely to contribute to a better understanding of these diseases by identifying causative genes and downstream signaling pathways that can be influenced pharmacologically. Genetic research should complement future work to identify the as yet unknown environmental risk factor(s) responsible for the development of autoimmune liver disease through interaction with genetic factors [24]. One of the greatest challenges in genetic epidemiological studies remains deriving a functional biological interpretation of the results from GWAS. In the following, I describe the current status from GWAS studies for AIH, PBC, and PSC and briefly outline what additional work I believe is promising to better understand the genetic component and its biological contribution.

## HLA-related genetic associations from GWAS

AIH, PBC, and PSC show a strong association with classical human leukocyte antigen genes (class I and II HLA genes; region on chromosome *6p21*), which is a common feature of autoimmune diseases, with HLA susceptibility variants usually having a much greater impact than any other risk variant in the genome [25]. Proteins encoded by HLA genes are expressed on cell surfaces and present processed antigens to immune cells, which then activate downstream immune processes. A GWAS is an association study involving several million single nucleotide polymorphisms (SNPs) to determine the contribution of genetic variants to disease susceptibility [26]. The first GWAS for AIH [27], PBC [28], and PSC [29] revealed genetic associations close to classical HLA susceptibility alleles discovered before the GWAS era [30]. Subsequent GWAS and genome-wide meta-analyses (GWMA) used HLA imputation methods to investigate a variety of HLA alleles as possible susceptibility alleles. HLA imputation is a method of deriving HLA types for patients and controls in GWAS studies by imputing (predicting) genotypes of HLA genes using regional SNPs and a SNP-HLA-allele reference panel for imputation [31]. In a first GWAS for AIH [27], involving 649 adult AIH patients and 13,436 controls, followed by replication in 451 patients and 4103 controls, the strongest genome-wide significant (*P* < 5 × 10^−8^) association signal for SNP rs2187668 was found at *6p21.32*, and HLA imputation assigned the SNP signals to HLA-DRB1*03:01, which is considered the primary AIH susceptibility allele (Table 1); HLA-DRB1*04:01 was identified as another independent (i.e., secondary) AIH susceptibility allele by conditional forward stepwise logistic regression analysis [32]. Recently, a meta-analysis of two GWAS study populations from China (1622 Chinese AIH type 1 patients and 10,466 population controls) identified a SNP association signal near *HLA-B* [33]. Results of GWAS and HLA fine-mapping for other populations as well as transethnic studies (i.e., studies across globally different study populations with largely different haplotype structure) are not available for AIH but would be of great importance to identify additional HLA susceptibility alleles and determine susceptibility to AIH across populations. In PBC, a total of 14 HLA alleles with genome-wide significance were identified in a HLA Immunochip fine-mapping study of 2861 PBC cases and 8514 controls of European ancestry [34], with four independent HLA association clusters for PBC identified by conditional logistic regression (Table 1). The first three independent HLA susceptibility alleles (clusters representatives) DQA1*04:01, DQB1*06:02 and DQB1*03:01 confirmed findings from serological studies [35–37], with the fourth allele DRB1*04:04 not previously associated with PBC. In another Immunochip HLA fine-mapping study of 676 Italian PBC cases and 1440 controls, three *DRB1* (DRB1*08, DRB1*11, DRB1*14) and one *DPB1* (DPB1*03:01) susceptibility cluster were identified through conditional analysis [38], although DRB1*14 and DPB1*03:01 did not meet the genome-wide significance threshold and are therefore not listed in Table 1. An HLA genotyping study of 1200 Japanese PBC patients and 1196 controls found a primary contribution of DQB1*06:04 and DQB1*03:01 to PBC susceptibility [39]. Subsequently, a GWAS and HLA fine mapping study of 1126 Han Chinese PBC patients and 1770 controls showed that *DRB1* (with DRB1*08:03) and/or *DQB1* (with DQB1*03:01) picked up most of the signals, with *DPB1* (DPB1*17:01) being an independent locus [40]. Interestingly, the protective DQB1*03:01 allele for PBC has been identified as a secondary association signal in populations of European origin and in Japan, whereas it is considered a primary association signal in the Han Chinese population. For a complete list of HLA susceptibility alleles for PBC, including alleles from candidate studies, see Gerussi et al. [41]. For PSC, five independent HLA association clusters (B*08:01; DQA*01:03; DQA*05:01; DRB1*15:01; DQA*01:01) were identified in an Immunochip fine-mapping study of 3789 PSC cases and 25,079 population controls [42]. In their combined stepwise regression analysis of HLA alleles and SNPs, HLA class II associations were consistent with previous studies [43, 44], with the exception of DQA1*01:01 which was newly added (Table 1). For a complete list of HLA susceptibility alleles for PSC from candidate studies up to 2013, see Mells et al. [45], although this list of candidate HLA alleles has not been expanded to include with new candidates from non-GWAS studies since the advent of several GWMA studies for PSC. Regression analysis can work with covariates and allows disentangling the HLA effect from confounding factors such as population stratification, sex, and others. However, conditional forward stepwise regression analysis has several methodological disadvantages. First, as the number of conditioning steps increases, so does the number of statistical tests. If there are *m* alleles in the region of interest, about k*m statistical tests are performed after *k* consecutive steps, which significantly increases the probability of a false positive result. Second, if *m* is large, perhaps close to the number of individuals in the GWAS study, and if a liberal significance threshold is used to include alleles at each step, the forward selection procedure becomes unstable and is too optimistic about the disease variation explained by the selected allele. For this reason, I have listed in Table 1 only statistically independent and genome-wide significant HLA susceptibility alleles (cluster representatives) that are also genome-wide significant after conditioning regression analysis. The identification (fine-mapping) of a complete set of potentially “causal” HLA alleles in the overall context of all class I and I genes requires the use of high-quality multi-ethnic reference panels from different genetic backgrounds [46, 47], highly accurate HLA type imputation algorithms [31], the study of non-additive and interaction effects [48], inclusion of ﻿amino acid alleles composing HLA alleles [49], and functional fine-mapping approaches [50–52]. Future HLA fine-mapping studies for AIH, PBC, and PSC therefore have the potential to further refine these signals from previous GWAS and HLA imputation studies.

**Table 1:** Statistically independent and genome-wide significant (*P* < 5 × 10^−8^) HLA susceptibility alleles identified by (hypothesis-free) GWAS/Immunochip analyses using HLA imputation models for classical HLA genes or by full HLA locus genotyping experiments. Only the representative allele of the HLA cluster from the respective publication is shown. HLA alleles from candidate gene studies are not listed. Effect direction refers to whether the minor allele increases or decreases the risk of disease. Secondary association signals were determined in the respective publication by stepwise logistic forward regression analysis, with the lead signals added as covariates. ^‡^SNP association signal near *HLA-B* gene was reported; no *HLA* allele association analysis was performed

Disease	HLA allele	Effect direction	Population	Lead/secondary signal	Reference
AIH	DRB1*03:01	Risk	Netherlands/Germany	Lead	De Boer et al. (2014) [27]
AIH	DRB1*04:01	Risk	Netherlands/Germany	Secondary	De Boer et al. (2014) [27]
AIH	*HLA-B*^‡^	Risk	China	Lead	Li et al. (2022) [33]
PBC	DQA1*04:01	Risk	UK	Lead	Liu et al. (2012) [34]
PBC	DQB1*06:02	Protective	UK	Secondary	Liu et al. (2012) [34]
PBC	DQB1*03:01	Protective	UK	Secondary	Liu et al. (2012) [34]
PBC	DRB1*04:04	Risk	UK	Secondary	Liu et al. (2012) [34]
PBC	DRB1*08	Risk	Italy	Lead	Invernizzi et al. (2012) [38]
PBC	DRB1*11	Protective	Italy	Secondary	Invernizzi et al. (2012) [38]
PBC	DQB1*06:04	Protective	Japan	Lead	Yasunami et al. (2017) [39]
PBC	DQB1*03:01	Protective	Japan	Secondary	Yasunami et al. (2017) [39]
PBC	DQB1*03:01	Protective	China	Lead	Wang et al. (2020) [40]
PBC	DPB1*17:01	Risk	China	Secondary	Wang et al. (2020) [40]
PBC	DRB1*08:03	Risk	China	Secondary	Wang et al. (2020) [40]
PSC	B*08:01	Risk	Europe and North America	Lead	Liu et al. (2013) [42]
PSC	DQA*01:03	Risk	Europe and North America	Secondary	Liu et al. (2013) [42]
PSC	DQA*05:01	Risk	Europe and North America	Secondary	Liu et al. (2013) [42]
PSC	DRB1*15:01	Risk	Europe and North America	Secondary	Liu et al. (2013) [42]
PSC	DQA*01:01	Risk	Europe and North America	Secondary	Liu et al. (2013) [42]

## Non-HLA-related genetic associations from GWAS

**Fig. 1:**
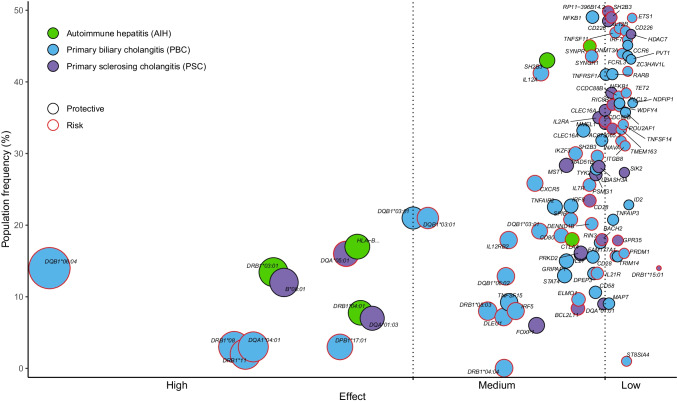
The polygenic landscape of HLA and non-HLA susceptibility variants for AIH, PBC, and PSC. Genome-wide significant (*P* < 5 × 10^−8^) HLA and non-HLA susceptibility variants were identified by GWAS and HLA imputation (Table 1) and GWAS meta-analyses (GWMA; Table 2), respectively. Susceptibility variants were broadly categorized as high effect (odds ratio [OR] ≥ 2 for risk variants and OR ≤ 0.5 for protective variants), medium effect (2 > OR ≥ 1.2 or 0.5 < OR ≤ 0.83), and low effect (1.2 > OR > 1.0 or 0.83 < OR < 1.0), with the position of the variant on the *x* axis (on a log scale) corresponding to the magnitude of the OR. The population frequency indicated on the *y* axis refers to the minor allele frequency (MAF) of the susceptibility variant in the general population. The size of the circles represents the effect size, with a green circle border representing statistically protective variants (OR < 1 for the minor allele) and a red border representing risk variants (OR > 1 for the minor allele). ^‡^For AIH: association signal near *HLA-B* is based on SNP data only, see also Table 1

As the number of GWAS studies for AIH, PBC, and PSC increased, it became clear that the effect sizes of non-HLA associations were much smaller compared with associations with HLA alleles. For AIH, a coding variant rs3184504 in *SH2B3* was identified as a susceptibility variant with genome-wide significance in study populations from the Netherlands and Germany [27]; two additional non-coding variants (rs72929257 near *CTLA4* and rs6809477 at *SYNPR*) were recently identified across two study populations from China [33]. For PBC, the largest GWMAs of European (Asian) case–control populations yielded 45 (12) loci with genome-wide significance, with a total of 55 genome-wide significant non-HLA susceptibility loci identified in one or the other GWMA [53]. GWMAs for PSC identified a total of 22 genome-wide significant non-HLA susceptibility loci in Europeans [42, 54, 55]; non-European as well as trans-ethnic GWMAs have not yet been performed for PSC. Table 2 summarizes all non-HLA susceptibility variants from GWMA studies for AIH, PBC, and PSC. For a review of non-HLA susceptibility variants for AIH, PBC, and PSC from monocentric studies, including candidate studies, see Engel et al. [56], Gerussi et al. [41], and Chung/Hirschfeld [57], respectively. For PBC, Bayesian fine-mapping was recently performed for the 55 non-HLA PBC susceptibility loci [53]. Bayesian methods are particularly well suited for fine-mapping of non-HLA loci to identify statistically “causal” sets of variants [58] and have been successfully used, for example, to fine-map inflammatory bowel disease (IBD) risk loci to single-variant resolution [59]. For 40 (9) of 55 non-HLA PBC susceptibility loci, the association signal was best explained by a single variant (posterior probability ≥ 0.5) across European and Asian (Asian only) populations (Table 2); for AIH and PSC, Bayesian fine-mapping for established non-HLA susceptibility regions remains to be performed. Chromosome X association analysis has unfortunately been neglected in most GWAS studies [60]. Recently, a chromosome X-wide association study for PBC identified a genome-wide significant locus at Xp11.23 (the locus includes the *GRIPAP1* gene; see Table 2) in East Asian PBC case–control study populations, which also shows an association signal (not yet genome-wide significant) across European and Asian PBC case–control study sets [61]. X-linked inheritance models in GWAS/GWMA for PSC and AIH thus have the potential to reveal additional genetic associations. Figure 1 summarizes the polygenic landscape of genome-wide significant HLA and non-HLA susceptibility variants (each of which was named in association with a nearby candidate gene) for AIH, PBC, and PSC.

**Table 2:** Genome-wide significant (*P* < 5 × 10^−8^) non-HLA susceptibility variants identified by two (hypothesis-free) GWAS for AIH and several genome-wide meta-analyses (GWMA) for PBC and PSC. Susceptibility variants for PBC and PSC from monocentric studies and studies with candidate gene are not listed. Variant: dbSNP name of the variant. Chromosome:position: human genome build hg37. Candidate gene: candidate gene from the respective publication. Fine-mapped to single variant: In cases where loci could be resolved to a single variant by Bayesian fine-mapping with high probability as causal (posterior probability > 50%), the name of the variant is indicated. NA: fine-mapping results not yet available

Disease	Variant	Chromosome:position	Candidate gene	Population	Fine-mapped to single variant	Reference
AIH	rs3184504	12:111,884,608	*SH2B3*	Netherlands/Germany	NA	De Boer et al. (2014) [27]
AIH	rs72929257	2:204,982,643	*CTLA4*	China	NA	Li et al. (2022) [33]
AIH	rs6809477	3:63,563,282	*SYNPR*	China	NA	Li et al. (2022) [33]
PBC	rs867436	1:2,523,723	*MMEL1*	European ancestry	rs867436	Cordell et al. (2021) [53]
PBC	rs6679356	1:67,820,194	*IL12RB2*	European ancestry	rs6679356	Cordell et al. (2021) [53]
PBC	rs10802191	1:117,065,083	*CD58*	European ancestry	rs10802191	Cordell et al. (2021) [53]
PBC	rs945635	1:157,670,290	*FCRL3*	European ancestry	rs945635	Cordell et al. (2021) [53]
PBC	rs12123169	1:197,780,966	*DENND1B*	European ancestry	rs12123169	Cordell et al. (2021) [53]
PBC	rs55734382	1:201,019,059	*INAVA*	European ancestry	rs55734382	Cordell et al. (2021) [53]
PBC	rs34655300	2:25,514,333	*DNMT3A*	European ancestry	rs891058	Cordell et al. (2021) [53]
PBC	rs859767	2:135,341,200	*TMEM163*	European ancestry	Several signals	Cordell et al. (2021) [53]
PBC	rs3771317	2:191,543,962	*STAT4*	European ancestry	Several signals	Cordell et al. (2021) [53]
PBC	rs9876137	3:16,961,265	*PLCL2*	European ancestry	rs9876137	Cordell et al. (2021) [53]
PBC	rs6550965	3:25,383,587	*RARB*	European ancestry	rs6550965	Cordell et al. (2021) [53]
PBC	rs2293370	3:119,219,934	*CD80*	European ancestry	rs2293370	Cordell et al. (2021) [53]
PBC	rs589446	3:159,733,527	*IL12A*	European ancestry	Several signals	Cordell et al. (2021) [53]
PBC	rs7674640	4:103,540,780	*NFKB1*	European ancestry	rs7674640	Cordell et al. (2021) [53]
PBC	rs7663401	4:106,128,954	*TET2*	European ancestry	rs7663401	Cordell et al. (2021) [53]
PBC	rs35467801	5:35,881,130	*IL7R*	European ancestry	rs35467801	Cordell et al. (2021) [53]
PBC	rs2546890	5:158,759,900	*IL12B*	European ancestry	rs2546890	Cordell et al. (2021) [53]
PBC	rs2327832	6:137,973,068	*TNFAIP3*	European ancestry	Several signals	Cordell et al. (2021) [53]
PBC	rs7805218	7:20,378,801	*ITGB8*	European ancestry	rs7805218	Cordell et al. (2021) [53]
PBC	rs60600003	7:37,382,465	*ELMO1*	European ancestry	rs60600003	Cordell et al. (2021) [53]
PBC	rs12531711	7:128,617,466	*IRF5*	European ancestry	Several signals	Cordell et al. (2021) [53]
PBC	rs370193557	7:138,729,543	*ZC3HAV1L*	European ancestry	rs370193557	Cordell et al. (2021) [53]
PBC	rs11390003	9:100,741,912	*TRIM14*	European ancestry	rs11390003	Cordell et al. (2021) [53]
PBC	rs7097397	10:50,025,396	*WDFY4*	European ancestry	rs7097397	Cordell et al. (2021) [53]
PBC	rs58523027	11:646,986	*IRF7*	European ancestry	Several signals	Cordell et al. (2021) [53]
PBC	rs11601860	11:64,110,422	*CCDC88B*	European ancestry	rs11601860	Cordell et al. (2021) [53]
PBC	rs12419634	11:111,239,365	*POU2AF1*	European ancestry	Several signals	Cordell et al. (2021) [53]
PBC	rs201150316	11:118,740,104	*CXCR5*	European ancestry	rs201150316	Cordell et al. (2021) [53]
PBC	rs1800693	12:6,440,009	*TNFRSF1A*	European ancestry	rs1800693	Cordell et al. (2021) [53]
PBC	rs35350651	12:111,907,431	*SH2B3*	European ancestry	rs35350651	Cordell et al. (2021) [53]
PBC	rs9533122	13:43,055,002	*TNFSF11*	European ancestry	rs9533122	Cordell et al. (2021) [53]
PBC	rs9591325	13:50,811,220	*DLEU1*	European ancestry	rs9591325	Cordell et al. (2021) [53]
PBC	rs3784099	14:68,749,927	*RAD51B*	European ancestry	rs3784099	Cordell et al. (2021) [53]
PBC	rs72699866	14:93,114,787	*RIN3*	European ancestry	Several signals	Cordell et al. (2021) [53]
PBC	rs59643720	14:103,564,807	*TNFAIP2*	European ancestry	Several signals	Cordell et al. (2021) [53]
PBC	rs9652601	16:11,174,365	*CLEC16A*	European ancestry	Several signals	Cordell et al. (2021) [53]
PBC	rs1119132	16:27,403,469	*IL21R*	European ancestry	rs1119132	Cordell et al. (2021) [53]
PBC	rs79577483	16:68,036,939	*DPEP3*	European ancestry	rs79577483	Cordell et al. (2021) [53]
PBC	rs11117432	16:86,019,271	*IRF8*	European ancestry	Several signals	Cordell et al. (2021) [53]
PBC	rs33938760	17:38,044,893	*IKZF3*	European ancestry	rs33938760	Cordell et al. (2021) [53]
PBC	rs1029464	17:44,149,348	*MAPT*	European ancestry	rs1029464	Cordell et al. (2021) [53]
PBC	rs1808094	18:67,526,026	*CD226*	European ancestry	rs1808094	Cordell et al. (2021) [53]
PBC	rs2304256	19:10,475,652	*TYK2*	European ancestry	rs2304256	Cordell et al. (2021) [53]
PBC	rs3745516	19:50,926,742	*SPIB*	European ancestry	Several signals	Cordell et al. (2021) [53]
PBC	rs137687	22:39,740,078	*SYNGR1*	European ancestry	Several signals	Cordell et al. (2021) [53]
PBC	rs10802190	1:117,061,384	*CD58*	China/Japan	rs10802190	Cordell et al. (2021) [53]
PBC	rs842349	2:135,342,452	*TMEM163*	China/Japan	rs842349	Cordell et al. (2021) [53]
PBC	rs11889341	2:191,943,742	*STAT4*	China/Japan	Several signals	Cordell et al. (2021) [53]
PBC	rs4675370	2:204,646,499	*CD28*	China/Japan	rs4675370	Cordell et al. (2021) [53]
PBC	rs12695386	3:119,209,027	*POGLUT1*	China/Japan	rs12695386	Cordell et al. (2021) [53]
PBC	rs230534	4:103,449,041	*NFKB1*	China/Japan	rs230534	Cordell et al. (2021) [53]
PBC	rs34463936	5:35,850,149	*LOC105374724*	China/Japan	rs34463936	Cordell et al. (2021) [53]
PBC	rs4709148	6:167,521,676	*CCR6*	China/Japan	rs4709148	Cordell et al. (2021) [53]
PBC	rs56211063	9:117,585,897	*TNFSF15*	China/Japan	Several signals	Cordell et al. (2021) [53]
PBC	rs4938534	11:111,275,133	*BTG4*	China/Japan	rs4938534	Cordell et al. (2021) [53]
PBC	rs480958	11:118,577,990	*LOC105369519*	China/Japan	Several signals	Cordell et al. (2021) [53]
PBC	rs12942330	17:37,939,839	*IKZF3*	China/Japan	rs12942330	Cordell et al. (2021) [53]
PBC	rs13416555	2:8,441,735	*ID2*	European + Asian ancestry	rs891058	Cordell et al. (2021) [53]
PBC	rs10581773	2:204,660,748	*CD28*	European + Asian ancestry	rs10581773	Cordell et al. (2021) [53]
PBC	rs60643069	5:100,238,073	*ST8SIA4*	European + Asian ancestry	rs141002831	Cordell et al. (2021) [53]
PBC	rs6874308	5:141,506,911	*NDFIP1*	European + Asian ancestry	rs10062349	Cordell et al. (2021) [53]
PBC	rs742108	6:106,582,920	*PRDM1*	European + Asian ancestry	Several signals	Cordell et al. (2021) [53]
PBC	rs968334	6:167,526,096	*CCR6*	European + Asian ancestry	rs3093024	Cordell et al. (2021) [53]
PBC	rs4733851	8:129,264,420	*PVT1*	European + Asian ancestry	several signals	Cordell et al. (2021) [53]
PBC	rs1322057	9:117,578,374	*TNFSF15*	European + Asian ancestry	rs6478109	Cordell et al. (2021) [53]
PBC	rs10893872	11:128,325,553	*ETS1*	European + Asian ancestry	rs10893872	Cordell et al. (2021) [53]
PBC	rs799469	14:35,444,425	*FAM177A1*	European + Asian ancestry	rs712315	Cordell et al. (2021) [53]
PBC	rs7059064	X:48,837,087	*GRIPAP1*	China/Japan	NA	Asselta et al. (2021) [61]
PSC	rs7556897	2:228,660,112	*AC073065.3*	European ancestry	NA	Ellinghaus et al. (2016) [54]
PSC	rs17032705	4:103,432,974	*NFKB1*	European ancestry	NA	Ellinghaus et al. (2016) [54]
PSC	rs12369214	12:107,198,611	*RIC8B*	European ancestry	NA	Ellinghaus et al. (2016) [54]
PSC	rs11649613	16:11,319,357	*RP11-396B14.2*	European ancestry	NA	Ellinghaus et al. (2016) [54]
PSC	rs3748816	1:2,526,746	*MMEL1*	European ancestry	NA	Ji et al. (2016) [55]
PSC	rs72837826	2:111,933,001	*BCL2L11*	European ancestry	NA	Ji et al. (2016) [55]
PSC	rs7426056	2:204,612,058	*CD28*	European ancestry	NA	Ji et al. (2016) [55]
PSC	rs3749171	2:241,569,692	*GPR35*	European ancestry	NA	Ji et al. (2016) [55]
PSC	rs3197999	3:49,721,532	*MST1*	European ancestry	NA	Ji et al. (2016) [55]
PSC	rs80060485	3:71,153,890	*FOXP1*	European ancestry	NA	Ji et al. (2016) [55]
PSC	rs13140464	4:123,499,745	*IL21*	European ancestry	NA	Ji et al. (2016) [55]
PSC	rs56258221	6:91,030,441	*BACH2*	European ancestry	NA	Ji et al. (2016) [55]
PSC	rs4147359	10:6,108,439	*IL2RA*	European ancestry	NA	Ji et al. (2016) [55]
PSC	rs663743	11:64,107,735	*CCDC88B*	European ancestry	NA	Ji et al. (2016) [55]
PSC	rs7937682	11:111,579,939	*SIK2*	European ancestry	NA	Ji et al. (2016) [55]
PSC	rs11168249	12:48,208,368	*HDAC7*	European ancestry	NA	Ji et al. (2016) [55]
PSC	rs3184504	12:111,884,608	*SH2B3*	European ancestry	NA	Ji et al. (2016) [55]
PSC	rs725613	16:11,169,683	*CLEC16A*	European ancestry	NA	Ji et al. (2016) [55]
PSC	rs1788097	18:67,543,688	*CD226*	European ancestry	NA	Ji et al. (2016) [55]
PSC	rs60652743	19:47,205,707	*PRKD2*	European ancestry	NA	Ji et al. (2016) [55]
PSC	rs2836883	21:40,466,744	*PSMG1*	European ancestry	NA	Ji et al. (2016) [55]
PSC	rs1893592	21:43,855,067	*UBASH3A*	European ancestry	NA	Ji et al. (2016) [55]

## SNP-based (co)-heritability

The proportion of genetic variance in liability (i.e., heritability explained by individual genetic variants for binary outcomes; additive model based on disease prevalence, relative risks, and allele frequencies [62]) for PBC explained by four major HLA alleles [34] (Table 1) and 26 independent genome-wide significant non-HLA susceptibility variants (subset from Table 2) was estimated to be 4.9% and 1.4%, respectively, which account together for 16.2% of total PBC heritability [34]. More recent calculations using the much larger PBC GWAS sets available today would be desirable, as would estimates for AIH. SNP-based heritability for PSC explained by 16 independent genome-wide significant loci (including major HLA alleles) account for 7.3% of total PSC heritability [42]; again, more up-to-date estimates would be desirable. The discrepancy between the variance caused by common SNPs and the expected heritability of AILDs from twin studies is referred to as missing heritability [63]. This gap in heritability can have several possible causes: Heritability from twin studies could be overestimated because common environmental factors or non-additive effects were not taken into account. On the other hand, part of the heritability could be due to genetic variants that have remained undetected so far, such as rare genetic variants or sex chromosome variants. Non-additive genetic effects, such as dominance effects ($\delta$ ^2^_SNP_) or additive-by-additive interaction effects ($\eta$ ^2^_SNP_; epistasis), might describe part of the disease heritability in AILDs. In a recent study of 70 (continuous) complex traits from the UK Biobank (with more than 60,000 individuals for each trait), the average epistatic variance across all traits ($\widehat{\eta }$ ^2^_SNP_ = 0.055) was estimated to be significantly higher than the average variance for dominance effects ($\widehat{\delta }$ ^2^_SNP_ = 0.001), but still significantly lower than the average variance for additive effects ($\widehat{h}$ ^2^_SNP_ = 0.208) [64]. Genome-wide interaction studies (GWIS) are therefore another interesting approach, but GWIS for PBC, PSC, and AIH would require many times the current sample size (which is difficult to realize) due to the exponential increase in statistical tests and extremely longer calculation times [65], although alternative computer architectures such as GPUs could help here [66, 67].

Because up to 9% and 7% of AIH patients have clinical overlap with PBC and PSC [27], respectively, it is reasonable to assume that there are shared genetic factors for AIH, PBC, and PSC [68], and also for other immune-mediated diseases, as shown by Immunochip studies for PBC and PSC [34, 38, 42]. In AIH, genetic risk is shared with type 1 diabetes for DRB1*04:01 [69] and systemic lupus erythematosus for DRB1*03:01 [70]. The association of AIH with the *SH2B3* locus has also been identified as a genetic risk factor for PSC and PBC; more specifically, even the same risk variant rs3184504 in *SH2B3* has been identified for AIH and PSC (see Table 1). Genetic relationships between disease pairs on a genome-wide level can be investigated by genome-wide genetic correlation analyses that quantify genome-wide SNP-based heritability (*h*_*g*_^2^_SNP_) in a bivariate model and provide information on potential coheritability between diseases [71–73]. Unfortunately, the shared genetics of AIH, PBC, and PSC have been studied only to a limited extent genome-wide and only partially with other immune-mediated comorbidities. When SNPs from the extended major histocompatibility complex (MHC) region (chromosome 6 region of 25–34 Mb including HLA genes) were excluded, a significant genetic correlation was observed between PSC and inflammatory bowel disease (ulcerative colitis, *r*_g_ = 0.29; Crohn’s disease, *r*_g_ = 0.04). For 196 fine-mapped regions of the Immunochip, the genetic correlation between PSC and ulcerative colitis (*r*_g_ = 0.64), PSC and Crohn’s disease (*r*_g_ = 0.35), and PSC and ankylosing spondylitis (*r*_g_ = 0.33) was highest compared with non-immune diseases [54]. To accurately identify possible shared causal variants in AIH-, PBC-, and PSC-associated regions, Bayesian tests of colocalization may be useful [74] and could provide an indication of whether there are common or independent causal variants for the same genomic regions. For example, six of 14 loci associated with both PSC and IBD showed strong evidence of a shared causal variant with UC, CD, or both [55]; colocalization analyses for AIH, PBC, and PSC could provide further insight into shared genetic structure. Future genome-wide comparisons between (worldwide) study populations with AIH, PBC, and PSC would provide the opportunity to identify the potentially shared landscape of AILDs.

## Polygenic risk scores

**Fig. 2:**
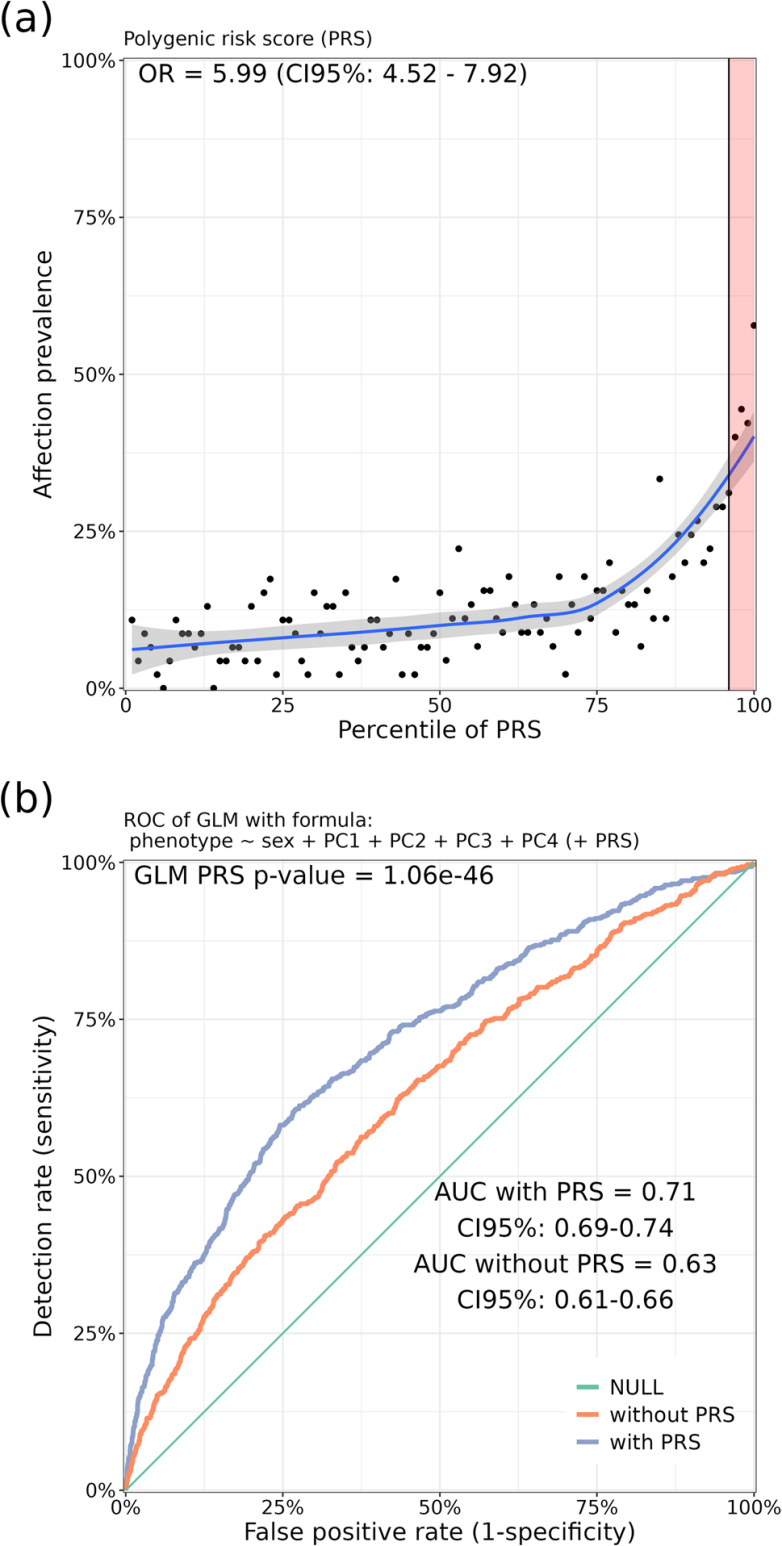
Risk gradient for PSC affection status according to the polygenic risk score (PRS) percentile and corresponding receiver operating characteristic curve (ROC) for a resulting (very weak) diagnostic test. **a** 100 groups of the test data set were derived according to the percentile of the PSC-specific PRS. The prevalence indicated on the *y* axis of the graph refers to the ratio of cases to controls in the genotyped sample. Odds ratio (OR) was calculated by comparing individuals with high PRS (top 5%) with the rest of the population (remaining 95%) in a logistic regression model adjusted for sex and the first four principal components (PCs) of ancestry from principal components analysis (PCA) with GWAS data. **b** Sensitivity: proportion of affected individuals with positive test results. False-positive rate (1—specificity): proportion of unaffected individuals with positive test results. AUC: area under the curve

Genome-wide SNP-based (co-)heritability estimation provides information on the proportion of (co-)heritability explained for (pairs of) diseases and measures pleiotropy (vertical and horizontal [75]) between diseases, but does not provide an estimate of individual patient risk based on genetic markers. Given the polygenic nature of AILDs (Fig. 1) and the fact that individual risk variants from GWAS/GWMA describe only a fraction of the heritability, a combined genetic burden across all genetic variants can be calculated to identify individuals at significant increased risk. A polygenic risk score (PRS) is an estimate of an individual’s genetic susceptibility to a disease calculated based on that individual's genotype profile and relevant data from GWAS. A study by Khera and colleagues [76] revived the topic of PRS for common complex diseases and showed for coronary artery disease (CAD), that a PRS identifies 20-times more individuals at comparable or greater risk than did previous studies for monogenic mutations. Therefore, identifying individuals with high (low) PRS in a population-based sample may provide an opportunity to identify those with the highest (lowest) genetic risk. However, the utility of PRS-based risk estimates for AIH, PBC, and PSC is limited by the small effect sizes of the identified susceptibility variants (see Fig. 1). Using genome-wide data from UK Biobank, Khera and colleagues showed that individuals in the top 5% of a PRS for CAD had a 3.34-fold risk [OR(CI95%) = 3.34(3.12–3.58)]; *P*_logistic_regression_ = 6.5 × 10^−264^] compared with the remaining 95% of the general population. To provide an estimate for PSC here (although our GWAS study data here is not a population-based sample), I calculated a PRS from the summary statistics of the most recent GWMA for PSC [55] and determined the distribution of the PRS for 628 GWMA-independent German PSC cases and 4,272 healthy controls (Methods). A PRS for PSC runs the risk of creating a mixed PRS for PSC and IBD, as patients with PSC have a highly increased incidence of IBD (called PSC with concomitant IBD or PSC-IBD); however, PSC-IBD has clinical differences from classical IBD [77] and appears to be genetically distinct from classical IBD phenotypes [54] (see below). Individuals in the top 5% of the PRS for PSC had a 5.99-fold risk [OR(CI95%) = 5.99(4.52–7.92); *P*_logistic_regression_ = 1.06 × 10^−46^ and adjusted for sex and genetic ancestry] compared with the remaining 95% of the general population (Fig. 2a). The OR should be interpreted as the factor by which the chance of developing the disease increases if a person has a positive PRS test result (here, being in the top 5%). However, with an OR = 5.99 and an underlying false positive rate (1-specificity) of 5%, the detection rate (sensitivity) is only 24% (Fig. 2b), resulting in an OAPR (odds of being affected given a positive result) of 1:2087, compared with the overall prevalence of 1:10,000 for PSC in the general population. On this basis, diagnostic genetic testing would be inappropriate. The fact that even a very high OR is associated with low predictive power of a diagnostic test may seem counterintuitive. It is largely explained by the fact that the genetic risk variants are widespread in the general population, so almost everyone can be affected by these causes, even if not everyone is or becomes ill because of the genetic burden. Therefore, the PRSs for AILDs in their current form are not suitable for diagnostic testing. On the other hand, for example, a possibly increased PRS for PSC in patients with AIH (compared with healthy controls) could indicate shared genetic risk factors (pleiotropy) between PSC and AIH, but an increased cross-disease PRS could be due to multiple causes such as diagnostic misclassification, molecular subtypes, or excessive comorbidity (collectively referred to as heterogeneity). Cross-locus correlation analyses of loci associated with disease B in cases of disease A (and vice versa) can help distinguish pleiotropy from heterogeneity. For PSC and IBD, for example, we have shown that PSC-IBD is likely to be a distinct disease at the genetic level, sharing some genetic factors with IBD, but genetically distinct from classical IBD phenotypes such as CD and UC [54].

## Genetically regulated expression and single cell analyses

Susceptibility variants identified by GWAS are often located at genomic positions with methylation, expression, and protein-quantitative trait loci (mQTLs, eQTLs, pQTLs), but it remains unclear whether this overlap is due to methylation, expression, and protein levels "mediating" genetic effects on disease. Cordell and colleagues applied Bayesian tests for colocalization between GWMA summary statistics of PBC and genome-wide mQTLs, eQTLs, and pQTLs data from large-scale consortium projects ALSPAC [78], GTEx [79], and INTERVAL [80] and suggested that the genetic architecture of PBC influences susceptibility to the disease primarily by affecting the regulation of expression of potentially causal genes [53]. To assess whether this might also be expected for PSC, I calculated the correlation between GWMA summary statistics of PSC [55] and summary statistics of large tissue-specific eQTL studies from GTEx using an approach developed by Yao et al. [81] called mediated expression score regression (MESC). I estimated that the heritability for PSC mediated by the cis genetic component of gene expression levels (*h*_*med*_^2^/*h*_*g*_^2^) averaged 38.4% for 48 tissues used in the GTEx project (Methods). This value of 38.4% for PSC is among the top of all disease-specific MESC values published in the work of Yao et al. who studied 42 diseases and human traits in the same 48 tissues, including ulcerative colitis with a similarly high published value of 38.2%. Therefore, it is hypothesized that in PBC and PSC (perhaps also in AIH), the gap between genetic approaches and the resulting disease phenotype can be reduced by the transcriptome. Because eQTL data from bulk tissues are thought to be a poor surrogate for eQTL data in causal cell types/contexts and little is known about the composition of intrahepatic immune cells and their contribution to disease pathogenesis, measurement of context-specific expression [82] and expression in single cells may allow the identification of genetic variants that impact key regulatory networks in AILDs [83]. Using single-cell RNA sequencing (scRNA-seq) techniques, Poch and colleagues generated the first atlas of intrahepatic T cells in PSC and identified a previously uncharacterized population of liver-resident CD4 + T cells that likely contribute to the pathogenesis of PSC [84]. Xiang and colleagues [85] developed a computational framework to integrate GWAS summary statistics with scRNA-seq data and revealed genetically modulated liver cell subpopulations for PBC. They found that cholangiocytes show significant enrichment with PBC-related genetic association signals, with the *ORMDL3* gene showing the highest expression level in cholangiocytes compared with other liver cells. Such combined genetics/single cell omics studies have the potential to identify the causative genes for AIH, PBC, and PSC in a disease-specific context.

## Genome-wide screenings for drug reuse

More than 25% of drugs entering clinical development fail because of lack of efficacy, but drugs with supportive genetic evidence are twice as likely to succeed in clinical development as drugs without supportive genetic evidence [86]. Thus, one potential approach is to test drugs with genetic support that have been successfully used in practice for other immune-mediated diseases for their transferability to AILDs. To this end, Cordell and colleagues have developed an elegant in-silico method for identifying drugs that can improve (or exacerbate) PBC in this prediction [53], highlighting the potential of genomic screening approaches for drug discovery and prediction of opposing drug effects in complex diseases. Briefly, they adapted a network-based approach to drug proximity screening from Guney et al. [87] for PBC candidate genes from GWAS risk loci by calculating a measure of proximity (z-score) between candidate genes and known drug targets (from agents stored in Drugbank), where a low *z*-score indicates recommended use of these agents (because an agent’s gene targets are closer to susceptibility genes than expected by chance) and a high *z*-score represents non-recommended use (because an agent’s targets are not closer to susceptibility risk genes than expected by chance). Major drugs predicted to improve PBC included ustekinumab, a monoclonal antibody against IL-12/23 used to treat psoriasis and Crohn's disease [88]; however, a proof-of-concept study has not shown benefit of ustekinumab for patients with PBC [89]. Major drugs that could exacerbate PBC included the pharmacologic interferons interferon alfa-2a and interferon beta-1b. The drugs already approved for PBC, fenofibrate, bezafibrate, and OCA were confirmed; interestingly, UDCA did not achieve a significant result, suggesting that genetics does not play a role in this case. A similar analysis would be desirable for AIH and PSC.

## Conclusion

A GWAS for AIH and several GWMAs for PBC and PSC have been successfully conducted and have identified a variety of genetic factors associated with AIH, PBC, and PSC. Some of these studies have already identified disease-causing variants by statistical fine-mapping and provided important biological insights into pathogenesis. Some statistical epidemiological approaches, such as statistical fine-mapping, chromosome X-wide association testing, and genome-wide screens for drug reuse, that have already been successfully performed in PBC, could also be applied to AIH and PSC. Large-scale cross-disease GWMAs to explore the shared genetic landscape of AIH, PBC, and PSC are still lacking. Merging genetic and statistical results with single-cell transcriptomic data from relevant cell types and liver tissue is likely to provide more accurate insights into the effects of genetic factors on liver cells and their immunological microenvironment.

## Software

PRS derivation: The LDPred2 software [90] (https://github.com/privefl/bigsnpr) was used to generate a PRS. The MESC software [81] (https://github.com/douglasyao/mesc) was used for estimating heritability mediated by assayed gene expression levels.

## References

[1] Boberg KM (2002). Prevalence and epidemiology of autoimmune hepatitis. Clin Liver Dis.

[2] Hennes EM, Zeniya M, Czaja AJ, Pares A, Dalekos GN, Krawitt EL, Bittencourt PL, Porta G, Boberg KM, Hofer H, Bianchi FB, Shibata M, Schramm C, Eisenmann de Torres B, Galle PR, McFarlane I, Dienes HP, Lohse AWG (2008) International autoimmune hepatitis, simplified criteria for the diagnosis of autoimmune hepatitis. Hepatology 48(1):169–76 10.1002/hep.22322 18537184

[3] Krawitt EL (2006). Autoimmune hepatitis. N Engl J Med.

[4] Pape S, Schramm C, Gevers TJ (2019). Clinical management of autoimmune hepatitis. United European Gastroenterol J.

[5] Gronbaek L, Vilstrup H, Pedersen L, Christensen K, Jepsen P (2018). Family occurrence of autoimmune hepatitis: a Danish nationwide registry-based cohort study. J Hepatol.

[6] Tanaka A, Leung PSC, Gershwin ME (2019). The genetics of primary biliary cholangitis. Curr Opin Gastroenterol.

[7] European Association for the Study of the Liver (2017) Electronic address, L. European Association for the Study of the, EASL Clinical Practice Guidelines: the diagnosis and management of patients with primary biliary cholangitis. J Hepatol 67(1):145–172 10.1016/j.jhep.2017.03.022 28427765

[8] Lleo A, Leung PSC, Hirschfield GM, Gershwin EM (2020). The pathogenesis of primary biliary cholangitis: a comprehensive review. Semin Liver Dis.

[9] Jones DE, Watt FE, Metcalf JV, Bassendine MF, James OF (1999). Familial primary biliary cirrhosis reassessed: a geographically-based population study. J Hepatol.

[10] Selmi C, Mayo MJ, Bach N, Ishibashi H, Invernizzi P, Gish RG, Gordon SC, Wright HI, Zweiban B, Podda M, Gershwin ME (2004). Primary biliary cirrhosis in monozygotic and dizygotic twins: genetics, epigenetics, and environment. Gastroenterology.

[11] Karlsen TH, Folseraas T, Thorburn D, Vesterhus M (2017). Primary sclerosing cholangitis - a comprehensive review. J Hepatol.

[12] Folseraas T, Melum E, Franke A, Karlsen TH (2011). Genetics in primary sclerosing cholangitis. Best Pract Res Clin Gastroenterol.

[13] Bansi D, Chapman R, Fleming K (1996). Antineutrophil cytoplasmic antibodies in chronic liver diseases: prevalence, titre, specificity and IgG subclass. J Hepatol.

[14] Chapman RW (1995). The enigma of anti-neutrophil antibodies in ulcerative colitis primary sclerosing cholangitis: important genetic marker or epiphenomenon?. Hepatology.

[15] Delle Monache M, Salvio A, Fiocca F, Basoli A, Ricci GL (1992) Primary sclerosing cholangitis: an analysis of 37 retrospective cases. Ital J Gastroenterol 24(9):485–8 1489978

[16] Bergquist A, Montgomery SM, Bahmanyar S, Olsson R, Danielsson A, Lindgren S, Prytz H, Hultcrantz R, Loof LA, Sandberg-Gertzen H, Almer S, Askling J, Ehlin A, Ekbom A (2008). Increased risk of primary sclerosing cholangitis and ulcerative colitis in first-degree relatives of patients with primary sclerosing cholangitis. Clin Gastroenterol Hepatol.

[17] Mieli-Vergani G, Vergani D, Czaja AJ, Manns MP, Krawitt EL, Vierling JM, Lohse AW, Montano-Loza AJ (2018). Autoimmune hepatitis. Nat Rev Dis Primers.

[18] Carbone M, Mells GF, Pells G, Dawwas MF, Newton JL, Heneghan MA, Neuberger JM, Day DB, Ducker SJ, Consortium UP, Sandford RN, Alexander GJ, Jones DE (2013) Sex and age are determinants of the clinical phenotype of primary biliary cirrhosis and response to ursodeoxycholic acid. Gastroenterology 144(3):560–569 e7; quiz e13–4 10.1053/j.gastro.2012.12.005 23246637

[19] Bahar R, Wong KA, Liu CH, Bowlus CL (2018). Update on new drugs and those in development for the treatment of primary biliary cholangitis. Gastroenterol Hepatol (N Y).

[20] Weiler-Normann C, Schramm C, Quaas A, Wiegard C, Glaubke C, Pannicke N, Moller S, Lohse AW (2013). Infliximab as a rescue treatment in difficult-to-treat autoimmune hepatitis. J Hepatol.

[21] Than NN, Hodson J, Schmidt-Martin D, Taubert R, Wawman RE, Botter M, Gautam N, Bock K, Jones R, Appanna GD, Godkin A, Montano-Loza AJ, Lammert F, Schramm C, Manns MP, Swain M, Burak KW, Adams DH, Hirschfield GM, Oo YH (2019). Efficacy of rituximab in difficult-to-manage autoimmune hepatitis: Results from the International Autoimmune Hepatitis Group. JHEP Rep.

[22] Gerussi A, Luca M, Cristoferi L, Ronca V, Mancuso C, Milani C, D’Amato D, O'Donnell SE, Carbone M, Invernizzi P (2020). New therapeutic targets in autoimmune cholangiopathies. Front Med (Lausanne).

[23] Bossen L, Gerussi A, Lygoura V, Mells GF, Carbone M, Invernizzi P (2018). Support of precision medicine through risk-stratification in autoimmune liver diseases - histology, scoring systems, and non-invasive markers. Autoimmun Rev.

[24] Karlsen TH, Chung BK (2015). Genetic risk and the development of autoimmune liver disease. Dig Dis.

[25] Horton R, Wilming L, Rand V, Lovering RC, Bruford EA, Khodiyar VK, Lush MJ, Povey S, Talbot CC, Wright MW, Wain HM, Trowsdale J, Ziegler A, Beck S (2004). Gene map of the extended human MHC. Nat Rev Genet.

[26] Tam V, Patel N, Turcotte M, Bosse Y, Pare G, Meyre D (2019). Benefits and limitations of genome-wide association studies. Nat Rev Genet.

[27] de Boer YS, van Gerven NM, Zwiers A, Verwer BJ, van Hoek B, van Erpecum KJ, Beuers U, van Buuren HR, Drenth JP, den Ouden JW, Verdonk RC, Koek GH, Brouwer JT, Guichelaar MM, Vrolijk JM, Kraal G, Mulder CJ, van Nieuwkerk CM, Fischer J, Berg T, Stickel F, Sarrazin C, Schramm C, Lohse AW, Weiler-Normann C, Lerch MM, Nauck M, Volzke H, Homuth G, Bloemena E, Verspaget HW, Kumar V, Zhernakova A, Wijmenga C, Franke L, Bouma G, Dutch Autoimmune Hepatitis Study G, LifeLines Cohort S, P. (2014) Study of health in, Genome-wide association study identifies variants associated with autoimmune hepatitis type 1. Gastroenterology 147(2): 443–52;e5 10.1053/j.gastro.2014.04.022 24768677

[28] Hirschfield GM, Liu X, Xu C, Lu Y, Xie G, Lu Y, Gu X, Walker EJ, Jing K, Juran BD, Mason AL, Myers RP, Peltekian KM, Ghent CN, Coltescu C, Atkinson EJ, Heathcote EJ, Lazaridis KN, Amos CI, Siminovitch KA (2009). Primary biliary cirrhosis associated with HLA, IL12A, and IL12RB2 variants. N Engl J Med.

[29] Karlsen TH, Franke A, Melum E, Kaser A, Hov JR, Balschun T, Lie BA, Bergquist A, Schramm C, Weismuller TJ, Gotthardt D, Rust C, Philipp EE, Fritz T, Henckaerts L, Weersma RK, Stokkers P, Ponsioen CY, Wijmenga C, Sterneck M, Nothnagel M, Hampe J, Teufel A, Runz H, Rosenstiel P, Stiehl A, Vermeire S, Beuers U, Manns MP, Schrumpf E, Boberg KM, Schreiber S (2010). Genome-wide association analysis in primary sclerosing cholangitis. Gastroenterology.

[30] Czaja AJ, Strettell MD, Thomson LJ, Santrach PJ, Moore SB, Donaldson PT, Williams R (1997). Associations between alleles of the major histocompatibility complex and type 1 autoimmune hepatitis. Hepatology.

[31] Naito T, Okada Y (2022). HLA imputation and its application to genetic and molecular fine-mapping of the MHC region in autoimmune diseases. Semin Immunopathol.

[32] Morris AP (2014). Fine mapping of type 2 diabetes susceptibility loci. Curr Diab Rep.

[33] Li Y, Sun Y, Liu Y, Wang B, Li J, Wang H, Zhang H, Wang X, Han X, Lin Q, Zhou Y, Hu L, Song Y, Bao J, Gong L, Sun M, Yuan X, Zhang X, Lian M, Xiao X, Miao Q, Wang Q, Li KK, Du S, Ma A, Li Y , Xu J, Tang S , Shi J, Xu Y, Yang L, Zhang J, Huang Z, Zhou L, Cui Y, Seldin MF, Gershwin ME, Yan H, Zou Z, Zuo X, Tang R, Ma X, Chinese AIHC (2022) Genome-wide meta-analysis identifies novel susceptibility loci for autoimmune hepatitis Type 1. Hepatology 10.1002/hep.32417 35184318

[34] Liu JZ, Almarri MA, Gaffney DJ, Mells GF, Jostins L, Cordell HJ, Ducker SJ, Day DB, Heneghan MA, Neuberger JM, Donaldson PT, Bathgate AJ, Burroughs A, Davies MH, Jones DE, Alexander GJ, Barrett JC, Sandford RN, Anderson CA, UKPBC Consortium, C. (2012) Wellcome Trust case control, dense fine-mapping study identifies new susceptibility loci for primary biliary cirrhosis. Nat Genet 44(10):1137–41 10.1038/ng.2395 PMC3459817 22961000

[35] Wassmuth R, Depner F, Danielsson A, Hultcrantz R, Loof L, Olson R, Prytz H, Sandberg-Gertzen H, Wallerstedt S, Lindgren S (2002). HLA class II markers and clinical heterogeneity in Swedish patients with primary biliary cirrhosis. Tissue Antigens.

[36] Donaldson PT, Baragiotta A, Heneghan MA, Floreani A, Venturi C, Underhill JA, Jones DE, James OF, Bassendine MF (2006). HLA class II alleles, genotypes, haplotypes, and amino acids in primary biliary cirrhosis: a large-scale study. Hepatology.

[37] Mullarkey ME, Stevens AM, McDonnell WM, Loubiere LS, Brackensick JA, Pang JM, Porter AJ, Galloway DA, Nelson JL (2005). Human leukocyte antigen class II alleles in Caucasian women with primary biliary cirrhosis. Tissue Antigens.

[38] Invernizzi P, Ransom M, Raychaudhuri S, Kosoy R, Lleo A, Shigeta R, Franke A, Bossa F, Amos CI, Gregersen PK, Siminovitch KA, Cusi D, de Bakker PI, Podda M, Gershwin ME, Seldin MF, Italian PBCGSG (2012) Classical HLA-DRB1 and DPB1 alleles account for HLA associations with primary biliary cirrhosis. Genes Immun 13(6):461–8 10.1038/gene.2012.17 PMC3423484 22573116

[39] Yasunami M, Nakamura H, Tokunaga K, Kawashima M, Nishida N, Hitomi Y, Nakamura M (2017). Principal contribution of HLA-DQ alleles, DQB1*06:04 and DQB1*03:01, to disease resistance against primary biliary cholangitis in a Japanese population. Sci Rep.

[40] Wang C, Zheng X, Tang R, Han C, Jiang Y, Wu J, Shao Y, Gao Y, Yu J, Hu Z, Zang Z, Zhao Y, Dai N, Liu L, Wu X, Nie J, Jiang B, Lin M, Li L, Wei Y, Li Y, Gong Y, Dai Y, Wang L, Ding N, Xu P, Chen S, Jiang P, Wang L, Qiu F, Wu Q, Zhang M, Jawed R, Chen R, Zhang Y, Shi X, Zhu Z, Pei H, Huang L, Tian Y, Zhang K, Qiu H, Zhao W, Gershwin ME, Chen W, Seldin MF, Liu X, Ma X, Sun L (2020). Fine mapping of the MHC region identifies major independent variants associated with Han Chinese primary biliary cholangitis. J Autoimmun.

[41] Gerussi A, Carbone M, Corpechot C, Schramm C, Asselta R, Invernizzi P (2021). The genetic architecture of primary biliary cholangitis. Eur J Med Genet.

[42] Liu JZ, Hov JR, Folseraas T, Ellinghaus E, Rushbrook SM, Doncheva NT, Andreassen OA, Weersma RK, Weismuller TJ, Eksteen B, Invernizzi P, Hirschfield GM, Gotthardt DN, Pares A, Ellinghaus D, Shah T, Juran BD, Milkiewicz P, Rust C, Schramm C, Muller T, Srivastava B, Dalekos G, Nothen MM, Herms S, Winkelmann J, Mitrovic M, Braun F, Ponsioen CY, Croucher PJ, Sterneck M, Teufel A, Mason AL, Saarela J, Leppa V, Dorfman R, Alvaro D, Floreani A, Onengut-Gumuscu S, Rich SS, Thompson WK, Schork AJ, Naess S, Thomsen I, Mayr G, Konig IR, Hveem K, Cleynen I, Gutierrez-Achury J, Ricano-Ponce I, van Heel D, Bjornsson E, Sandford RN, Durie PR, Melum E, Vatn MH, Silverberg MS, Duerr RH, Padyukov L, Brand S, Sans M, Annese V, Achkar JP, Boberg KM, Marschall HU, Chazouilleres O, Bowlus CL, Wijmenga C, Schrumpf E, Vermeire S, Albrecht M, Consortium U-P, International IBDGC, Rioux JD, Alexander G, Bergquist A, Cho J, Schreiber S, Manns MP, Farkkila M, Dale AM, Chapman RW, Lazaridis KN, International PSCSG, Franke A, Anderson CA, Karlsen TH (2013) Dense genotyping of immune-related disease regions identifies nine new risk loci for primary sclerosing cholangitis. Nat Genet 45(6):670–5 10.1038/ng.2616 PMC3667736 23603763

[43] Schrumpf E, Fausa O, Forre O, Dobloug JH, Ritland S, Thorsby E (1982). HLA antigens and immunoregulatory T cells in ulcerative colitis associated with hepatobiliary disease. Scand J Gastroenterol.

[44] Spurkland A, Saarinen S, Boberg KM, Mitchell S, Broome U, Caballeria L, Ciusani E, Chapman R, Ercilla G, Fausa O, Knutsen I, Pares A, Rosina F, Olerup O, Thorsby E, Schrumpf E (1999). HLA class II haplotypes in primary sclerosing cholangitis patients from five European populations. Tissue Antigens.

[45] Mells GF, Kaser A, Karlsen TH (2013). Novel insights into autoimmune liver diseases provided by genome-wide association studies. J Autoimmun.

[46] Degenhardt F, Wendorff M, Wittig M, Ellinghaus E, Datta LW, Schembri J, Ng SC, Rosati E, Hubenthal M, Ellinghaus D, Jung ES, Lieb W, Abedian S, Malekzadeh R, Cheon JH, Ellul P, Sood A, Midha V, Bk T, Wong SH, Schreiber S, Yamazaki K, Kubo M, Boucher G, Rioux J, Lenz TL, Brant SR, Franke A (2018) Construction and benchmarking of a multi-ethnic reference panel for the imputation of HLA class I and II alleles. Hum Mol Genet 10.1093/hmg/ddy443 PMC6548229 30590525

[47] Luo Y, Kanai M, Choi W, Li X, Sakaue S, Yamamoto K, Ogawa K, Gutierrez-Arcelus M, Gregersen PK, Stuart PE, Elder JT, Forer L, Schonherr S, Fuchsberger C, Smith AV, Fellay J, Carrington M, Haas DW, Guo X, Palmer ND, Chen YI, Rotter JI, Taylor KD, Rich SS, Correa A, Wilson JG, Kathiresan S, Cho MH, Metspalu A, Esko T, Okada Y, Han B, NT-Of PM Consortium, McLaren PJ, Raychaudhuri S (2021) A high-resolution HLA reference panel capturing global population diversity enables multi-ancestry fine-mapping in HIV host response. Nat Genet 53(10):1504–1516 10.1038/s41588-021-00935-7 PMC8959399 34611364

[48] Lenz TL, Deutsch AJ, Han B, Hu X, Okada Y, Eyre S, Knapp M, Zhernakova A, Huizinga TW, Abecasis G, Becker J, Boeckxstaens GE, Chen WM, Franke A, Gladman DD, Gockel I, Gutierrez-Achury J, Martin J, Nair RP, Nothen MM, Onengut-Gumuscu S, Rahman P, Rantapaa-Dahlqvist S, Stuart PE, Tsoi LC, van Heel DA, Worthington J, Wouters MM, Klareskog L, Elder JT, Gregersen PK, Schumacher J, Rich SS, Wijmenga C, Sunyaev SR, de Bakker PI, Raychaudhuri S (2015). Widespread non-additive and interaction effects within HLA loci modulate the risk of autoimmune diseases. Nat Genet.

[49] Hu X, Deutsch AJ, Lenz TL, Onengut-Gumuscu S, Han B, Chen WM, Howson JM, Todd JA, de Bakker PI, Rich SS, Raychaudhuri S (2015). Additive and interaction effects at three amino acid positions in HLA-DQ and HLA-DR molecules drive type 1 diabetes risk. Nat Genet.

[50] Aguiar VRC, Cesar J, Delaneau O, Dermitzakis ET, Meyer D (2019). Expression estimation and eQTL mapping for HLA genes with a personalized pipeline. PLoS Genet.

[51] Gutierrez-Arcelus M, Baglaenko Y, Arora J, Hannes S, Luo Y, Amariuta T, Teslovich N, Rao DA, Ermann J, Jonsson AH, NT-OfPM Consortium, Navarrete C, Rich SS, Taylor KD, Rotter JI, Gregersen PK, Esko T, Brenner MB, Raychaudhuri S (2020) Allele-specific expression changes dynamically during T cell activation in HLA and other autoimmune loci. Nat Genet 52(3):247–253 10.1038/s41588-020-0579-4 PMC7135372 32066938

[52] Yamamoto F, Suzuki S, Mizutani A, Shigenari A, Ito S, Kametani Y, Kato S, Fernandez-Vina M, Murata M, Morishima S, Morishima Y, Tanaka M, Kulski JK, Bahram S, Shiina T (2020). Capturing differential allele-level expression and genotypes of all classical HLA loci and haplotypes by a new capture RNA-Seq method. Front Immunol.

[53] Cordell HJ, Fryett JJ, Ueno K, Darlay R, Aiba Y, Hitomi Y, Kawashima M, Nishida N, Khor SS, Gervais O, Kawai Y, Nagasaki M, Tokunaga K, Tang R, Shi Y, Li Z, Juran BD, Atkinson EJ, Gerussi A, Carbone M, Asselta R, Cheung A, de Andrade M, Baras A, Horowitz J, Ferreira MAR, Sun D, Jones DE, Flack S, Spicer A, Mulcahy VL, Byan J, Han Y, Sandford RN, Lazaridis KN, Amos CI, Hirschfield GM, Seldin MF, Invernizzi P, Siminovitch KA, Ma X, Nakamura M, Mells GF, Consortia PBC, Canadian PBCC, Chinese PBCC, Italian PBCSG, Japan PBCGC, Consortium UP, Consortium U-P (2021) An international genome-wide meta-analysis of primary biliary cholangitis: novel risk loci and candidate drugs. J Hepatol 75(3):572–581

[54] Ellinghaus D, Jostins L, Spain SL, Cortes A, Bethune J, Han B, Park YR, Raychaudhuri S, Pouget JG, Hubenthal M, Folseraas T, Wang Y, Esko T, Metspalu A, Westra HJ, Franke L, Pers TH, Weersma RK, Collij V, D'Amato M, Halfvarson J, Jensen AB, Lieb W, Degenhardt F, Forstner AJ, Hofmann A, IBDGC International, C. International Genetics of Ankylosing Spondylitis, P.S.C.S.G. International, C. Genetic Analysis of Psoriasis, E. Psoriasis Association Genetics, S. Schreiber, U. Mrowietz, B.D. Juran, K.N. Lazaridis, S. Brunak, A.M. Dale, R.C. Trembath, S. Weidinger, M. Weichenthal, E. Ellinghaus, J.T. Elder, J.N. Barker, O.A. Andreassen, D.P. McGovern, T.H. Karlsen, J.C. Barrett, M. Parkes, M.A. Brown, Franke A (2016) Analysis of five chronic inflammatory diseases identifies 27 new associations and highlights disease-specific patterns at shared loci. Nat Genet 48(5):510–518 10.1038/ng.3528 PMC4848113 26974007

[55] Ji SG, Juran BD, Mucha S, Folseraas T, Jostins L, Melum E, Kumasaka N, Atkinson EJ, Schlicht EM, Liu JZ, Shah T, Gutierrez-Achury J, Boberg KM, Bergquist A, Vermeire S, Eksteen B, Durie PR, Farkkila M, Muller T, Schramm C, Sterneck M, Weismuller TJ, Gotthardt DN, Ellinghaus D, Braun F, Teufel A, Laudes M, Lieb W, Jacobs G, Beuers U, Weersma RK, Wijmenga C, Marschall HU, Milkiewicz P, Pares A, Kontula K, Chazouilleres O, Invernizzi P, Goode E, Spiess K, Moore C, Sambrook J, Ouwehand WH, Roberts DJ, Danesh J, Floreani A, Gulamhusein AF, Eaton JE, Schreiber S, Coltescu C, Bowlus CL, Luketic VA, Odin JA, Chopra KB, Kowdley KV, Chalasani N, Manns MP, Srivastava B,Mells G, Sandford RN, Alexander G, Gaffney DJ, Chapman RW, Hirschfield GM, de Andrade M, Consortium U-P, IBDGC International, P.S.C.S.G. International, Rushbrook SM, Franke A, Karlsen TH, Lazaridis KN, Anderson CA (2017) Genome-wide association study of primary sclerosing cholangitis identifies new risk loci and quantifies the genetic relationship with inflammatory bowel disease. Nat Genet 49(2):269–273 10.1038/ng.3745 PMC5540332 27992413

[56] Engel B, Laschtowitz A, Janik MK, Junge N, Baumann U, Milkiewicz P, Taubert R, Sebode M (2021). Genetic aspects of adult and pediatric autoimmune hepatitis: A concise review. Eur J Med Genet.

[57] Chung BK, Hirschfield GM (2017). Immunogenetics in primary sclerosing cholangitis. Curr Opin Gastroenterol.

[58] Schaid DJ, Chen W, Larson NB (2018). From genome-wide associations to candidate causal variants by statistical fine-mapping. Nat Rev Genet.

[59] Huang H, Fang M, Jostins L, Umicevic Mirkov M, Boucher G, Anderson CA, Andersen V, Cleynen I, Cortes A, Crins F, D'Amato M, Deffontaine V, Dmitrieva J, Docampo E, Elansary M, Farh KK, Franke A, Gori AS, Goyette P, Halfvarson J, Haritunians T, Knight J, Lawrance IC, Lees CW, Louis E, Mariman R, Meuwissen T, Mni M, Momozawa Y, Parkes M, Spain SL, Theatre E, Trynka G, Satsangi J, van Sommeren S, Vermeire S, Xavier RJ, C International inflammatory bowel disease genetics, Weersma RK, Duerr RH, Mathew CG, Rioux JD, McGovern DPB, Cho JH, Georges M, Daly MJ, Barrett JC (2017) Fine-mapping inflammatory bowel disease loci to single-variant resolution. Nature 547(7662):173–178 10.1038/nature22969 PMC5511510 28658209

[60] Konig IR, Loley C, Erdmann J, Ziegler A (2014). How to include chromosome X in your genome-wide association study. Genet Epidemiol.

[61] Asselta R, Paraboschi EM, Gerussi A, Cordell HJ, Mells GF, Sandford RN, Jones DE, Nakamura M, Ueno K, Hitomi Y, Kawashima M, Nishida N, Tokunaga K, Nagasaki M, Tanaka A, Tang R, Li Z, Shi Y, Liu X, Xiong M, Hirschfield G, Siminovitch KA, USPBCC Canadian A, Italian PBCGSG, Consortium U-P, Japan PBCGC, Carbone M, Cardamone G, Duga S, Gershwin ME, Seldin MF, Invernizzi P X (2021) Chromosome contribution to the genetic architecture of primary biliary cholangitis. Gastroenterology 160(7):2483–2495 e26 10.1053/j.gastro.2021.02.061 PMC8169555 33675743

[62] So HC, Gui AH, Cherny SS, Sham PC (2011). Evaluating the heritability explained by known susceptibility variants: a survey of ten complex diseases. Genet Epidemiol.

[63] Genin E (2020). Missing heritability of complex diseases: case solved?. Hum Genet.

[64] Hivert V, Sidorenko J, Rohart F, Goddard ME, Yang J, Wray NR, Yengo L, Visscher PM (2021). Estimation of non-additive genetic variance in human complex traits from a large sample of unrelated individuals. Am J Hum Genet.

[65] Wei WH, Hemani G, Haley CS (2014). Detecting epistasis in human complex traits. Nat Rev Genet.

[66] Hemani G, Theocharidis A, Wei W, Haley C (2011). EpiGPU: exhaustive pairwise epistasis scans parallelized on consumer level graphics cards. Bioinformatics.

[67] Wienbrandt L, Kassens JC, Ellinghaus D (2021). SNPInt-GPU: Tool for Epistasis Testing with Multiple Methods and GPU Acceleration. Methods Mol Biol.

[68] Boberg KM, Chapman RW, Hirschfield GM, Lohse AW, Manns MP, Schrumpf E, G. (2011) International autoimmune hepatitis, overlap syndromes: the International Autoimmune Hepatitis Group (IAIHG) position statement on a controversial issue. J Hepatol 54(2):374–85 10.1016/j.jhep.2010.09.002 21067838

[69] Erlich H, Valdes AM, Noble J, Carlson JA, Varney M, Concannon P, Mychaleckyj JC, Todd JA, Bonella P, Fear AL, Lavant E, Louey A, Moonsamy P, Type 1 Diabetes Genetics C (2008) HLA DR-DQ haplotypes and genotypes and type 1 diabetes risk: analysis of the type 1 diabetes genetics consortium families. Diabetes 57(4):1084–92 10.2337/db07-1331 PMC4103420 18252895

[70] Morris DL, Taylor KE, Fernando MM, Nititham J, Alarcon-Riquelme ME, Barcellos LF, Behrens TW, Cotsapas C, Gaffney PM, Graham RR, Pons-Estel BA, Gregersen PK, Harley JB, Hauser SL, Hom G, International MHC, Autoimmunity Genetics N, Langefeld CD, Noble JA, Rioux JD, Seldin MF, Systemic Lupus Erythematosus Genetics C, Criswell LA, Vyse TJ (2012) Unraveling multiple MHC gene associations with systemic lupus erythematosus: model choice indicates a role for HLA alleles and non-HLA genes in Europeans. Am J Hum Genet 91(5):778–93 10.1016/j.ajhg.2012.08.026 PMC3487133 23084292

[71] Lee SH, Yang J, Goddard ME, Visscher PM, Wray NR (2012). Estimation of pleiotropy between complex diseases using single-nucleotide polymorphism-derived genomic relationships and restricted maximum likelihood. Bioinformatics.

[72] Yang J, Lee SH, Goddard ME, Visscher PM (2011). GCTA: a tool for genome-wide complex trait analysis. Am J Hum Genet.

[73] BK Bulik-Sullivan, Loh PR, Finucane HK, Ripke S, Yang J, Schizophrenia Working Group of the Psychiatric Genomics C, Patterson N, Daly MJ, Price AL, Neale BM (2015) LD Score regression distinguishes confounding from polygenicity in genome-wide association studies. Nat Genet 47(3):291–5 10.1038/ng.3211 PMC4495769 25642630

[74] Giambartolomei C, Vukcevic D, Schadt EE, Franke L, Hingorani AD, Wallace C, Plagnol V (2014). Bayesian test for colocalisation between pairs of genetic association studies using summary statistics. PLoS Genet.

[75] van Rheenen W, Peyrot WJ, Schork AJ, Lee SH, Wray NR (2019). Genetic correlations of polygenic disease traits: from theory to practice. Nat Rev Genet.

[76] Khera AV, Chaffin M, Aragam KG, Haas ME, Roselli C, Choi SH, Natarajan P, Lander ES, Lubitz SA, Ellinor PT, Kathiresan S (2018). Genome-wide polygenic scores for common diseases identify individuals with risk equivalent to monogenic mutations. Nat Genet.

[77] de Vries AB, Janse M, Blokzijl H, Weersma RK (2015). Distinctive inflammatory bowel disease phenotype in primary sclerosing cholangitis. World J Gastroenterol.

[78] Relton CL, Gaunt T, McArdle W, Ho K, Duggirala A, Shihab H, Woodward G, Lyttleton O, Evans DM, Reik W, Paul YL, Ficz , Ozanne SE, Wipat A, Flanagan K, Lister A, Heijmans BT, Ring SM, Davey Smith G (2015) Data resource profile: accessible resource for integrated epigenomic Studies (ARIES). Int J Epidemiol 44(4):1181–90 10.1093/ije/dyv072 PMC5593097 25991711

[79] G.T. Consortium (2013). The genotype-tissue expression (GTEx) project. Nat Genet.

[80] Sun BB, Maranville JC, Peters JE, Stacey D, Staley JR, Blackshaw J, Burgess S, Jiang T, Paige E, Surendran P, Oliver-Williams C, Kamat MA, Prins BP, Wilcox SK, Zimmerman ES, Chi A, Bansal N, Spain SL, Wood AM, Morrell NW, Bradley JR, Janjic N, Roberts DJ, Ouwehand WH, Todd JA, Soranzo N, Suhre K, Paul DS, Fox CS, Plenge RM, Danesh J, Runz H, Butterworth AS (2018). Genomic atlas of the human plasma proteome. Nature.

[81] Yao DW, O'Connor LJ, Price AL, Gusev A (2020). Quantifying genetic effects on disease mediated by assayed gene expression levels. Nat Genet.

[82] Strober BJ, Elorbany R, Rhodes K, Krishnan N, Tayeb K, Battle A, Gilad Y (2019). Dynamic genetic regulation of gene expression during cellular differentiation. Science.

[83] van der Wijst MGP, Brugge H, de Vries DH, Deelen P, Swertz MA, LifeLines S, Cohort B, Consortium L, Franke (2018) Single-cell RNA sequencing identifies celltype-specific cis-eQTLs and co-expression QTLs. Nat Genet 50(4):493–497 10.1038/s41588-018-0089-9 PMC5905669 29610479

[84] Poch T, Krause J, Casar C, Liwinski T, Glau L, Kaufmann M, Ahrenstorf AE, Hess LU, Ziegler AE, Martrus G, Lunemann S, Sebode M, Li J, Schwinge D, Krebs CF, Franke A, Friese MA, Oldhafer KJ, Fischer L, Altfeld M, Lohse AW, Huber S, Tolosa E, Gagliani N, Schramm C (2021). Single-cell atlas of hepatic T cells reveals expansion of liver-resident naive-like CD4(+) T cells in primary sclerosing cholangitis. J Hepatol.

[85] Xiang B, Deng C, Qiu F, Li J, Li S, Zhang H, Lin X, Huang Y, Zhou Y, Su J, Lu M, Ma Y (2021). Single cell sequencing analysis identifies genetics-modulated ORMDL3(+) cholangiocytes having higher metabolic effects on primary biliary cholangitis. J Nanobiotechnology.

[86] Nelson MR, Tipney H, Painter JL, Shen J, Nicoletti P, Shen Y, Floratos A, Sham PC, Li MJ, Wang J, Cardon LR, Whittaker JC, Sanseau P (2015). The support of human genetic evidence for approved drug indications. Nat Genet.

[87] Guney E, Menche J, Vidal M, Barabasi AL (2016). Network-based in silico drug efficacy screening. Nat Commun.

[88] Hedin CRH, Sonkoly E, Eberhardson M, Stahle M (2021). Inflammatory bowel disease and psoriasis: modernizing the multidisciplinary approach. J Intern Med.

[89] G.M. Hirschfield, M.E. Gershwin, R. Strauss, M.J. Mayo, C. Levy, B. Zou, J. Johanns, I.P. Nnane, B. Dasgupta, K. Li, C. Selmi, H.U. Marschall, D. Jones, K. Lindor, P.S. Group (2016). Ustekinumab for patients with primary biliary cholangitis who have an inadequate response to ursodeoxycholic acid: a proof-of-concept study. Hepatology.

[90] Prive F, Arbel J, Vilhjalmsson BJ (2020) LDpred2: better, faster, stronger, Bioinformatics 10.1093/bioinformatics/btaa1029 PMC8016455 33326037

